# Innovation for improved hand hygiene: Field testing the Autarky handwashing station in collaboration with informal settlement residents in Durban, South Africa

**DOI:** 10.1016/j.scitotenv.2021.149024

**Published:** 2021-11-20

**Authors:** Catherine Sutherland, Eva Reynaert, Rebecca C. Sindall, Michel E. Riechmann, Fanelesibonge Magwaza, Juri Lienert, Sibongile Buthelezi, Duduzile Khumalo, Sifiso Dhlamini, Eberhard Morgenroth, Kai M. Udert

**Affiliations:** a University of KwaZulu Natal, School of Built Environment and Development Studies, 4041 Durban, South Africa; b Eawag, Swiss Federal Institute of Aquatic Science and Technology, 8600 Dübendorf, Switzerland; c ETH Zürich, Institute of Environmental Engineering, 8093 Zürich, Switzerland; d University of KwaZulu Natal, WASH R&D Centre, 4041 Durban, South Africa

**Keywords:** Community participation, Hand hygiene, WASH, Technology field test, Water recycling, Transdisciplinary research

## Abstract

Safe and accessible water services for hand hygiene are critical to human health and well-being. However, access to handwashing facilities is limited in cities in the Global South, where rapid urbanisation, service backlogs, lack of infrastructure and capacity, and water scarcity impact on the ability of local governments to provide them. Community participation and the co-production of knowledge in the development of innovative technologies, which are aligned with Water, Sanitation and Hygiene (WASH) principles, can lead to more sustainable and socially-acceptable hand hygiene systems. This paper presents the outcomes of the testing of the Autarky handwashing station, a technology that provides onsite treatment and recycling of handwashing water, in an informal settlement in Durban, South Africa. The transdisciplinary research approach adopted enabled the participation of multiple stakeholders with different knowledge systems in the framing, testing and evaluation of the system. The process of co-producing knowledge, as well as the outcomes of the testing, namely high levels of functionality and social acceptability of the technology, supported the WASH principles. The evaluation revealed that the Autarky handwashing station is a niche intervention that improved access to safe and appealing handwashing facilities in an informal settlement. Its novel design, socially desirable features, reliability and ability to save water increased its acceptance in the community. The testing of the system in a real-world context revealed the value of including communities in knowledge production processes for technology innovation. Further work is required to ensure that real-time monitoring of system function is feasible before such systems can be implemented at larger scale.

## Highlights

• Access to handwashing facilities is limited in cities in the Global South.
• The field-tested handwashing station addresses both social and environmental goals.
• The system is a niche intervention that supplements state-provided water services.
• Knowledge co-production achieves greater acceptability of innovative systems.
• Participation in service delivery improves state-citizen relations and builds capacity.

## Graphical abstract

**Figure:**
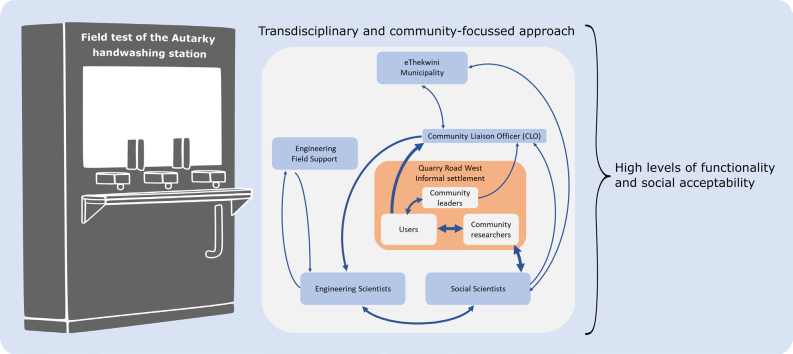

## 1 Introduction

The provision of safe, accessible, reliable and dignified water, sanitation and hygiene (WASH) services is a major global concern. Addressing WASH challenges aligns with the Sustainable Development Goals (SDGs), particularly SDG 3 (good health and well-being), SDG 6 (access to water and sanitation for all), and SDG 11 (sustainable cities and communities). Handwashing with soap and water is widely recognised as being essential in reducing disease transmission. However, in 2018, only 28% of people in the least developed countries had access to handwashing facilities with water and soap (United Nations, 2020). Safe and accessible handwashing systems have become critical in the COVID-19 pandemic, as one of the main strategies to reduce transmission is handwashing with soap. The pandemic has highlighted the lack of availability of water for basic hygiene practices in informal settlements, schools and rural areas in the Global South (Staddon et al., 2020).

The responsibility for providing water for hand hygiene varies across the world. In cities in the Global South, local governments face numerous challenges in providing access to safe and accessible water for the urban poor. These include increasing service backlogs due to rapid urbanisation and natural population increase; growing informality in cities; water scarcity, exacerbated by climate change; lack of human and financial resources to deliver services; and failures in service provision due to a lack of community engagement in their development (Maphela and Cloete, 2019; Taing et al., 2019; Tapela, 2015; van Vliet et al., 2011). As a result, innovation in water provision technologies, which is cognisant of these challenges, is required to improve access to WASH services and, ultimately, meet the SDGs. One such example is the Autarky handwashing station (AHWS), a technology that provides onsite recycling of handwashing water without the need for external water input (www.autarky.ch).

This paper presents the results of the field-testing of the AHWS for six months in Quarry Road West informal settlement in Durban, South Africa, using a transdisciplinary (Td) research approach. The field-testing comprised both the technical testing of the AHWS, and a community-centred social assessment, with all stakeholders framing the problems to be addressed (Bhaskar et al., 2010; Max-Neef, 2005; Pohl et al., 2017; Sutherland et al., 2021; You et al., 2020; Ziervogel et al., 2021). This approach recognised the importance of integrating knowledge from engineering and social sciences, and including the beneficiary community in the evaluation of the technology. Stakeholders from Eawag, the University of KwaZulu-Natal (WASH R&D Centre and School of Built Environment & Development Studies), eThekwini Municipality Water and Sanitation Unit (EWS) and the development and engineering firm Khanyisa Projects, co-produced knowledge jointly with the informal settlement community. This helped ensure that community voices formed a core part of the testing, and could inform the outcomes, shaping the future development of the technology.

## 2 Background

### 2.1 Innovative handwashing technologies

A range of technologies have been developed that enable handwashing in places that lack access to a piped water supply. Tippy taps are simple, economical handwashing stations, made with commonly available materials. They work by using the foot to press a lever, which tips water out from a raised container (Mbakaya et al., 2020). Non-governmental organizations and private companies (e.g., GIZ, Oxfam, Lixil Group) have developed a range of low-cost prefabricated handwashing stations, most of them for public settings. All these solutions require manual refilling of a tank with water, and thus require a safe source of water nearby, as no treatment of the water takes place in the systems. Additionally, these systems pose the risk of (re-)growth of pathogens if the water is stored for long periods. The water used for handwashing is disposed of to the ground or using a soak pit. There are only few technologies that support the recycling of water for handwashing or similar uses. Gassie et al. (2019) have developed a recycling shower station for remote emergency response. However, the technology is limited to usage for emergency periods, and has, as yet, only been tested in the laboratory. Some technologies are capable of filtering and recycling shower water over the course of a single showering event, with the additional benefit of decreasing energy consumption (e.g., Orbital Systems shower). However, these systems start with fresh water for each showering event, and thus do not allow for continued recycling of the water.

The AHWS, developed by the water research institute Eawag, in collaboration with the design office EOOS, is a promising technology, as it provides onsite recycling of handwashing water without the need for external water input (www.autarky.ch). The system can thus provide safe water in places that do not have sufficient water resources, or that lack infrastructure to distribute clean water and collect the wastewater. The AHWS is also relevant in contexts where green technologies are being supported, and where existing service provision needs to be supplemented.

The technology housed at the back of the AHWS is an on-site water recycling system called the Water Wall, which treats and recycles water for handwashing or toilet flushing without the need for external water input. As the Water Wall does not need any on-site infrastructure other than an electric power supply, it can be set up quickly and flexibly, potentially also for short periods. The system has been described in detail in Reynaert et al. (2020) and an overview of the technology is presented in the Supplementary information (SI A). Key features of the Water Wall are low power demand, robust operation, low maintenance requirements, and no consumables required during the treatment.

Prior to the testing at Quarry Road West informal settlement, an AHWS was field-tested in a public park in Zurich, Switzerland, where it provided safe and appealing handwashing water for up to 148 daily usages (Reynaert et al., 2020). The testing of the AHWS in Durban included an assessment of both social acceptance and technical functionality of the system. The technology was evaluated using principles for Water, Sanitation and Health (WASH) interventions as the assessment criteria.

### 2.2 Principles for Water Sanitation and Health (WASH) interventions

A set of WASH principles were developed from the literature to assess the outcomes of the field testing of the AHWS (Table 1). The WASH principles stress the importance of the social and governance dimensions of improved hygiene and sanitation, with an emphasis on community participation and understanding the social context, and do not focus only on the technical systems used to provide them.

**Table 1:** Guidelines for Water, Sanitation and Hygiene (WASH) interventions developed by the Swiss Red Cross (2014), and complemented with WASH principle C (Quality in design and functionality).

WASH principle	Importance
A. Meet Water Quality	To ensure the safety and acceptability of the technology by the community, it is essential that all the relevant water quality standards are adequately met.
B. Promote understanding of the community context	Building community trust is a critical aspect of the acceptance of innovative WASH technologies. Understanding the contextual background of each community provides relevant insight into socio-economic, environmental, cultural, political, and institutional relationships of communities.
C. Quality in design and functionality	To ensure the design of the system meets a range of human needs and is functional, and that the system is technically robust and reliable.
D. Empower and promote collaborative engagement and participation of stakeholders	The sustainability and successful implementation of WASH interventions in any community rest on the level of engagement and participation of different stakeholders (i.e., local government, civic groups, and other relevant private actors).
E. Promote equality	Poverty and inequality is a critical global challenge. It is thus essential to introduce a WASH intervention that is equally accessible, affordable, and beneficial to all people regardless of class or gender.

Collectively, the planning, design and implementation of WASH interventions aim to promote sustainability by considering and integrating the social, economic, environmental and governance dimensions of the WASH principles, to meet the SDGs, in particular contexts.

### 2.3 Social context of the testing

The eThekwini Municipal Area, or Durban, with its administrative entity eThekwini Municipality, is home to 3.7 million people, with 26% living in informal settlements (eThekwini Municipality, 2019). The eThekwini Water and Sanitation Unit (EWS) is mandated to provide water and sanitation services across the municipal area. The lack of adequate services in informal settlements in South Africa is a major challenge and results in frequent service protests, as people demand their constitutional right to basic services (Bond and Mottiar, 2018; Tapela, 2015). In Durban, informal settlement residents access water for hand hygiene mainly through communal tap points and Communal Ablution Blocks (CABs), provided by eThekwini Municipality's incremental services programme (eThekwini Municipality, 2019). CABs are prefabricated containers, which have flush toilets, handwash basins and showers, as well as large laundry basins at the back of the containers. The CABs are usually located on the edges of informal settlements, due to space constraints and the need to be close to bulk sewer infrastructure. These units are therefore not always located within 200 m of households, which national legislation stipulates is the maximum distance someone should travel to access water and sanitation. The development of innovative and affordable water and sanitation technologies is a priority in Durban's informal settlement upgrading programme (Odili and Sutherland, forthcoming).

**Fig. 1:**
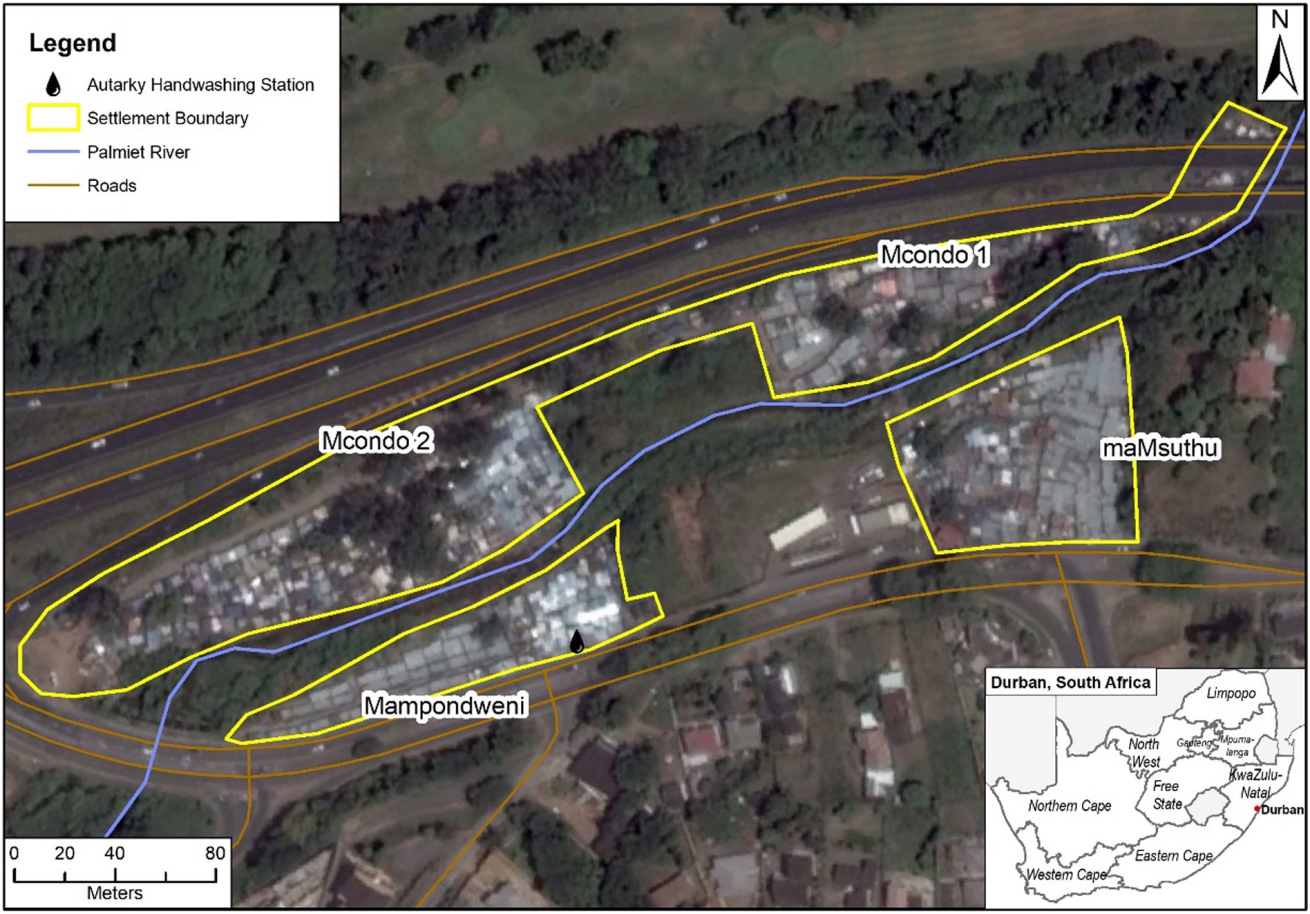
Quarry Road West informal settlement (Source: map produced by Michela du Sart, EduAction, Google Earth image: 2017).

The AHWS was tested in one of Durban's 566 informal settlements, Quarry Road West informal settlement, established in 1987. The settlement comprises of approximately 1100 households in four sections located on the floodplain of the Palmiet River (Mazeka et al., 2019) (Fig. 1). It is a highly precarious, dense settlement, with limited access to basic services, poor waste removal, and prone to flooding, fires and falling trees. The Palmiet River rises very quickly after storm events and floods its lower reaches, where the informal settlement is located, resulting in the loss of informal structures along the river banks (Sutherland, 2020). The settlement was devastated by a major flood on 21 April 2019, five days after the AHWS had been opened in the settlement, which influenced the outcomes of the testing process.

The participation of residents of Quarry Road West informal settlement in the Palmiet Catchment Rehabilitation Project (PCRP), as well as ongoing research conducted in the settlement through a partnership between researchers from the School of Built Environment and Development Studies (UKZN), the municipality and the community, has built a platform of social learning and knowledge co-production (Sutherland et al., 2019). This platform supported the co-production of knowledge from the outset of the testing and evaluation process of the AHWS, as it meant informal residents had experience in engaging in research processes.

## 3 Methodology

In February 2019, the technology was introduced to the community and an engagement and pre-evaluation process was initiated. This phase ran until April 2019, when the AHWS was installed in the community and opened for use (17 April 2020). The AHWS was tested for three months and a post evaluation survey was conducted at the end of July 2019.

### 3.1 Ethics statement

The methodologies used for this study were approved by the Research Ethics Committee at UKZN (approval numbers BE/045/19 and HSS/0153/019).

### 3.2 Implementation design and operation of the Autarky handwashing station

**Fig. 2:**
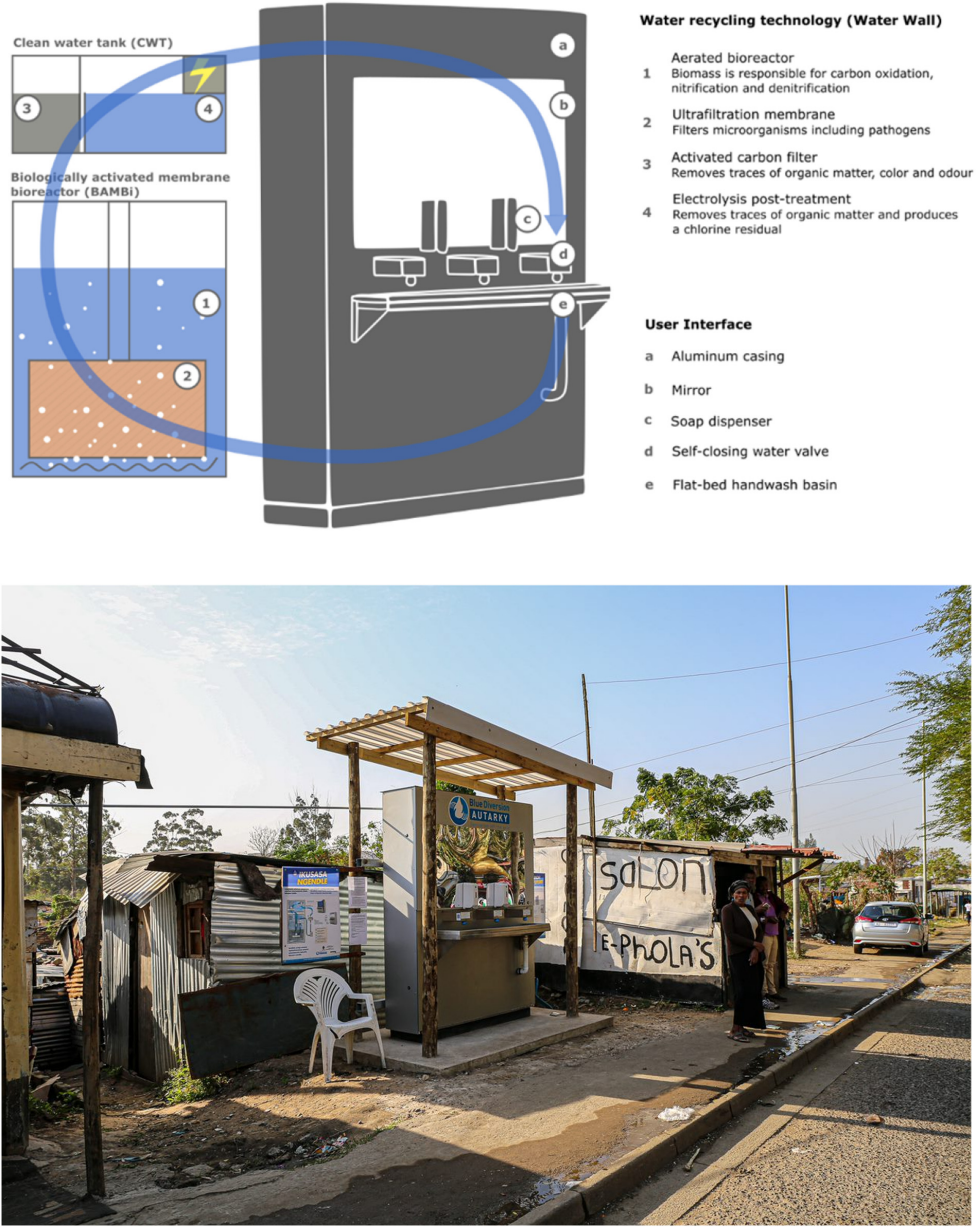
Top: schematic representation of the Autarky hand washing station (AHWS), with the frontend user interface and backend water recycling technology (Water Wall). Water was treated and recycled in a multi-barrier system with four treatment stages (bioreactor, ultrafiltration membrane, activated carbon filter, electrolysis post-treatment). Bottom: the AHWS in Quarry Road West informal settlement.

The prototype used in this testing consisted of the frontend user interface and the backend water recycling technology (Fig. 2). More detailed descriptions of the AHWS are presented in Reynaert et al. (2020).

The frontend was designed by the Austrian design office EOOS. It consisted of an aluminum casing covered by a large mirror at eye level. Users could wash their hands at three water valves (The Drop, ADED, Geneva, Switzerland). Two foaming soap dispenser (AlpineX Foam Soap Dispenser, Alpine Professional AG, Niederbipp, Switzerland) provided biodegradable soap. The used water was collected in a custom-made steel handwash basin, from where it entered the water treatment system.

For use in a public setting, signage and constructive means were used to manage residents' use of the system and to prevent them from drinking the water. The constructive means comprised (i) a solid casing impeding access to critical parts, (ii) the use of self-closing water valves to avoid dripping taps, and (iii) the implementation of a flat-bed hand wash basin minimizing the distance between the basin and the taps to avoid users drinking from the taps or bottling water. Signage on how to use the system was attached to the mirror. Additionally, each tap was labelled with “do not drink the water” stickers. Community members were also informed by the Community Liaison Officer (CLO), field engineer and community leaders that they should not drink the water. The tap design, the signage and information that passed through community networks, ensured that no one drank the water. Two posters located next to the AHWS provided information on the technical functioning of the system and on the motivation for testing it in a field trial (including details of a contact person). All information was provided in English and isiZulu.

### 3.3 Operation and monitoring

The CLO served as a local caretaker, checking the basic functioning of the station and the cleanliness on a daily basis. Additionally, a field engineer was on site two times per week to take water samples for laboratory analyses and to check that all system parts worked properly. The recycled water had to meet requirements for the chemical oxygen demand (COD), pH, residual chlorine, *E. coli* concentrations, turbidity and total suspended solids (TSS) defined by EWS (see Table 2) in order to receive the municipality's permission to test the AHWS in the field. Details on the methods used for water quality monitoring can be found in the Supplementary information (SI B).

**Table 2:** Water quality: comparison of field testing results to the eThekwini Water and Sanitation (EWS) Unit's requirements for the use of recycled water. MPN: most probable number.

Parameter	Unit	EWS requirements	Field-testing results
Chemical oxygen demand (COD)	mg/L	50 (avg) 150 (max)	9.1 (avg, *n* = 17) 36 (max)
pH	–	6–9	7.1–8.3 (*n* = 15)
Residual chlorine	mg/L	>0.5 (avg)	1.9 (avg, *n* = 24)
*E. coli*	MPN/100 mL	1 (avg) 10 (max)	<1 (avg, *n* = 18) <1 (max)
Turbidity	NTU	5 (avg) 10 (max)	0.44 (avg, *n* = 14) 0.83 (max)
Total suspended solids (TSS)	mg/L	10 (avg) 30 (max)	0.4 (avg, n = 1) 0.4 (max)

### 3.4 Social assessment

#### 3.4.1 Site selection and initial engagements

The Quarry Road West informal settlement was selected for (i) being representative of informal settlements in South Africa, and for having (ii) well-established research relationships with the university and eThekwini Municipality, as well as (iii) internal participatory governance structures, which support decision making in the community. Because of the Td approach adopted, the social assessment team ensured that the leadership of the community, and through their structures, community members, were consulted to determine if they were willing to test the AHWS in their community.

A focus group was held in the settlement on 4 February 2019 to present the idea of testing the AHWS in the settlement to the community. At this meeting, attended by team members from UKZN, Khanyisa Projects and the technology developers, community members asked questions about the technology and how it functioned, which proved useful to the project team. Community members were concerned about the safety of the water and wanted to understand details of how the system worked. They wanted to know how it inactivated pathogens and what safety mechanisms had been included in the system to ensure it would shut down should there be a malfunction. These safety mechanisms are now being developed and tested further in the laboratories at Eawag, partly as a result of the input received from the Quarry Road West informal community.

The social assessment team proposed that community members should experience using the AHWS before making the decision about testing it in their community. The community leadership from Quarry Road West informal settlement were invited to visit UKZN, where the AHWS was being tested in the university community. A workshop was held on campus which included an explanation of the functioning of the system by a field engineer, and the use of the AHWS.

Based on the focus group and the visit to UKZN, the community leadership, in consultation with the community, agreed to test the AHWS in Quarry Road West informal settlement. The location for the AHWS was selected by the community, considering the site requirements of the project team. The site needed to have (i) a footprint on flat ground, (ii) high foot traffic, (iii) access to an electrical connection, (iv) be easy to access for maintenance, and (v) be relatively secure. The AHWS was located along a main road in a place where the movement of people converges because of access to transport (taxi and bus stops) and spaza shops.^3^ It was also located in a space that did not impact on households in the settlement and was between a spaza shop and barber shop, which the community leaders stated would improve the security and maintenance of the system. The knowledge provided by community members proved to be invaluable in the siting of the AHWS, as it was well situated from a user perspective and had high visibility.

#### 3.4.2 Baseline survey, focus group discussion and post-testing survey

**Fig. 3:**
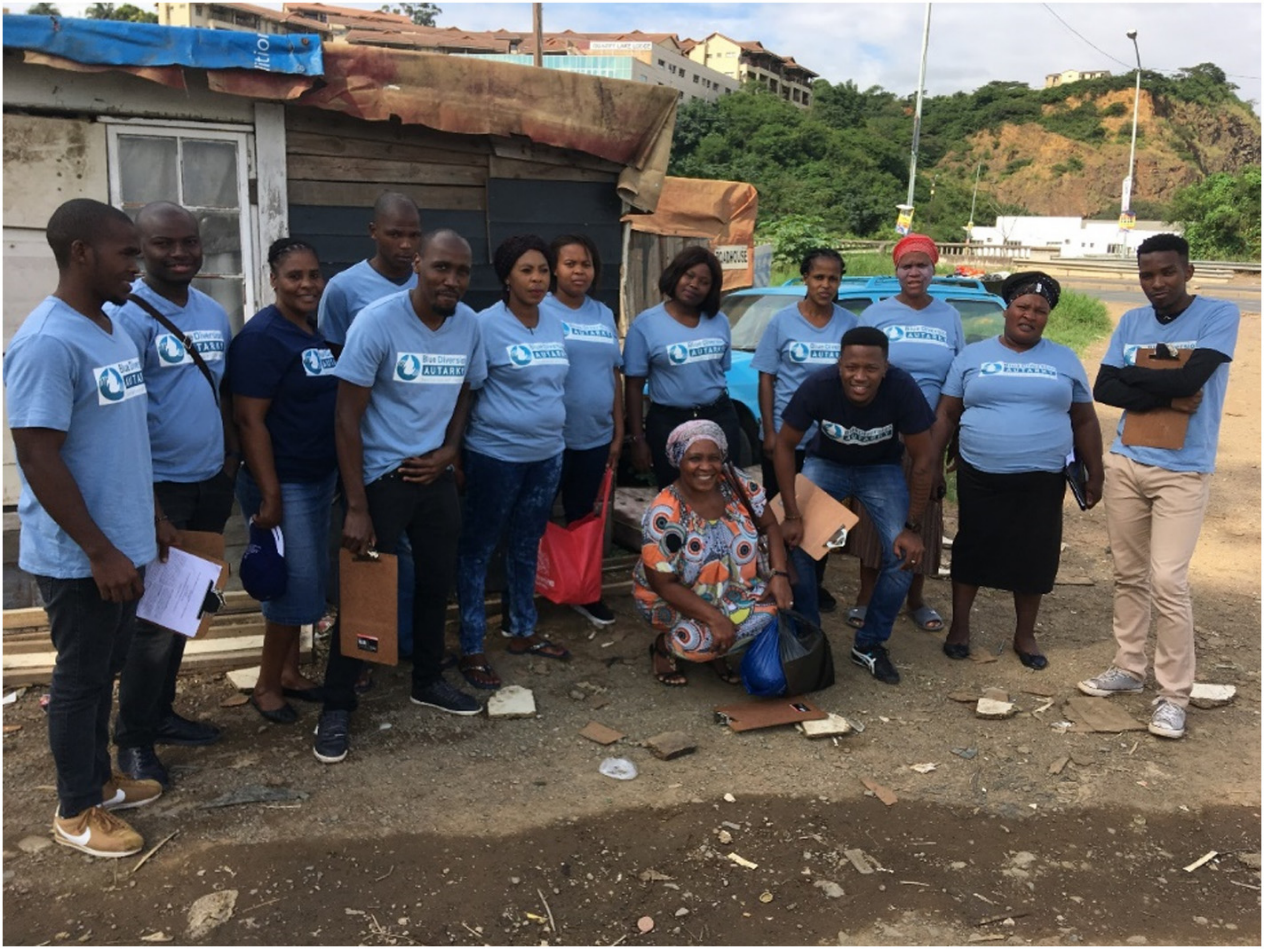
The UKZN and community-based researchers (Photograph taken by Catherine Sutherland 07/04/2019, permission for use of photograph provided by all participants).

Prior to the installation of the AHWS, a survey of 108 households (n_a_) was conducted by UKZN social scientists and twelve trained community-based researchers (7 to 12 April 2019). The sample size was 10% of the settlement population. The survey comprised of both open- and closed-ended questions. Households were sampled using clustered systematic random sampling, with 27 households selected from each of the four sections. The survey determined residents' access to water and sanitation services and their perceptions and attitudes towards them. The researchers wore Autarky-branded T-shirts to promote the AHWS, to legitimize and acknowledge their position as they conducted the research, and to encourage residents to use the system (Fig. 3). The community-based researchers reported that this raised awareness about the new technology and made the research partnership between university and community-based researchers visible (Community based researcher 2, Focus group, 03/05/2019).

During the testing period, a focus group was held on 3 May 2019 to assess the performance of the AHWS in the settlement. This data contributed to understanding the impact, acceptability, and satisfaction of the AHWS while it was still on site.

The post-test survey, which included both open- and closed-ended questions, was undertaken in July 2019, where 71 households (n_b_) that had used the AHWS were surveyed. A further 20 households who had not used the AHWS were surveyed, which meant the total sample for the post-evaluation survey was 91 households, as saturation had been reached. Households were selected using clustered systematic random sampling, with 23 households selected from each section. The survey assessed residents' experiences of using the system and their acceptance of it. Respondents could provide more than one answer to many of the questions, and so provided a wide diversity of responses. The subscript refers to each sample (n_a_: pre-test or n_b_: post-test). In cases where only a sub-set of respondents answered the questions, the percentage of the number that responded was calculated out of the sub-set of the sample (n_a1;_ n_a2_). The questionnaire data was coded and analysed using descriptive statistics in SPSS.

## 4 Results

This section presents the results of the Td research process used to evaluate the technical functioning and social acceptability of the AHWS, and the extent to which it is aligned with the WASH principles.

### 4.1 Water quality requirements were met

The technical functioning of the AHWS is described in detail in Reynaert et al. (2020). The water quality was compared to quality requirements set by EWS for the use of recycled water (Table 2). In terms of chemical water quality, the average and maximum COD, turbidity and TSS met the local requirements, and the pH was within the required range. In terms of hygienic water quality, the average chlorine concentration exceeded the requirements and no *E. coli* were detected in the treated water. Overall, the water was clear, colourless and had no smell.

Other than a weekly water refill to compensate for the losses due to evaporation and water removed on people's hands, and supplementation of sodium chloride (table salt) for chlorine production, the AHWS functioned without the need for regular maintenance. However, the electrolysis recirculation pump broke down on two occasions (days 19 and 63 of the field test) and required replacement. The AHWS was closed to the public from day 63 to 65, until the damaged pump was replaced. Apart from this closedown, water was available throughout the testing.

Nearly all users (97%) indicated that the water in the AHWS was clear. The clarity of the water was explained in more detail by 13% of respondents, who said that it looked like natural or normal water, and that it was clean. Almost all the users stated that the water did not smell (97%). Most users (82%) were happy and willing to use recycled water, with 8% unwilling to use it, 5% uncertain, and 5% did not respond. Most respondents (87%) stated they did not have any concerns about the safety of using recycled water in the AHWS, as they were satisfied with evidence of its safety (33%), the system had a filter (22%), it saved water (22%), it was clean (5%), and was only used for handwashing (5%). The main concern of the sub-group who felt the water was not safe (12% of users) was related to the potential of pathogens (“germs”) being present in the water (21% of the sub-group). Others (13% of the sub-group) stated that it was acceptable, as it was only used for hand washing, and 4% stated that once they got used to using it, they were less concerned about safety issues.

Almost all the residents that participated in the survey (97%) indicated that saving water by using treated recycled water for handwashing is an important need in Quarry Road West informal settlement. There was a high response (82%) by those who participated in the study as to why they thought this was important (Table 3). Overall, more than half of the respondents related the need for water recycling to water scarcity.

**Table 3:** Reasons why water recycling is important to residents in Quarry Road West informal settlement (multiple responses possible).

Category	% of respondents (n_a1_ = 58)	Example answers
Water scarcity	57%	“Water is a scarce resource” “It helps to prevent water shortages in the future”
Hygiene promotion	28%	“It helps us to stay clean” “It promotes hygiene”
Water availability	5%	“Sometimes we have no water in the CABs to wash our hands”
Saving money	5%	“It saves the municipality from paying water for us”

### 4.2 Autarky handwashing station aligned with community context and community needs

**Fig. 4:**
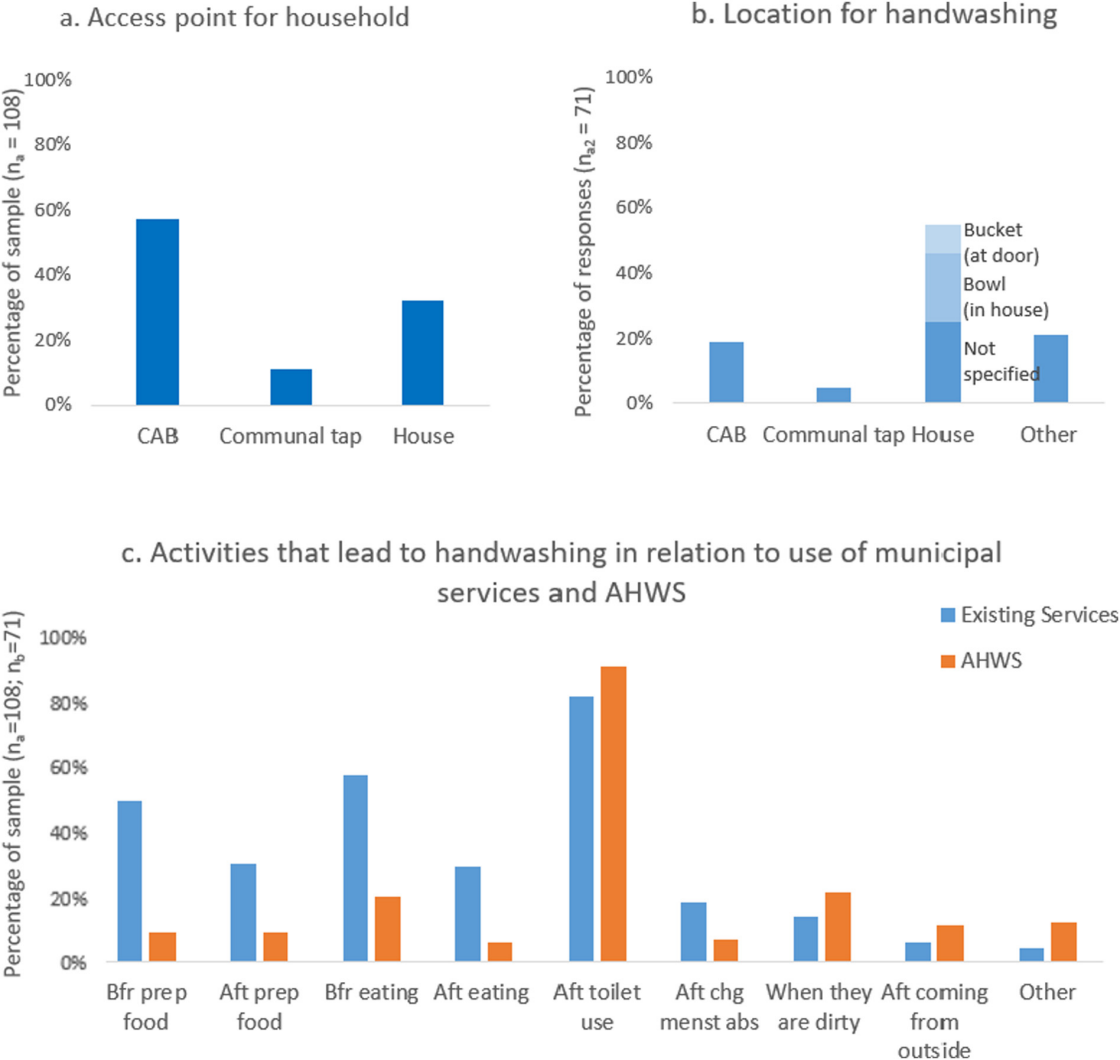
Access to water in Quarry Road West informal settlement prior to the installation of the Autarky handwashing station (AHWS) (a and b) and a comparison of handwashing practices prior to and after installation (c) (multiple answers possible). CAB: communal ablution block.

In analysing how well the AHWS aligned with the community context and needs, it was important to understand hygiene practices in Quarry Road West informal settlement prior to the installation of the AHWS. Residents of Quarry Road West informal settlement have access to water through communal taps, CABs and a few self-installed, illegal household taps (Fig. 4a). The water quality provided by EWS is universal and of a high standard throughout these access points. Respondents indicated that they had good access to water from communal taps and the CABs, with 86% reporting that water was always available in the CABs, 10% stated that sometimes water was not available and 3% replied that it was never available, while 1% did not respond. Almost all residents (99%) stated that they always had access to water to wash their hands, with the majority washing their hands in their homes (Fig. 4b). Apart from the sanitation facility provided in the CABs, 40% of the respondents valued the CABs for the provision of water used for both hygiene and household activities.

Fig. 4c compares the activities that led to handwashing using municipal water services prior to the installation of the AHWS and handwashing activities undertaken in relation to the AHWS after its installation. The data reveals that the AHWS, due to its function, location and convenience, was used less in relation to food preparation, as this type of handwashing was done inside the household, but was used more after using the toilet, when hands were dirty, when eating food, and on returning to the settlement from ‘outside’. Quarry Road West informal residents wash their hands regularly, with 94% stating they washed their hands every day. The majority of respondents wash their hands at home, using water, which has been stored in a bucket, bowl or other container (Fig. 3b). Most respondents washed their hands after toilet use and through their interactions with food, particularly before preparing food and before eating (Fig. 4c).

Many respondents stated that soap is important in handwashing to prevent the spread of disease and for good health, with 86% indicating that they used soap when they washed their hands, and 9% stating that they did not use soap. A majority of respondents (84%) said that they always have soap available to wash their hands. However, residents also stated that they do not regularly use soap to wash their hands in the CABs (69%), as soap is not available there. Only 25% of residents stated that they washed their hands with soap in the CABs. When they did wash their hands with soap, they reported that they had brought the soap to the CAB themselves (69%), with only 10% indicating that the municipality had provided it.

The AHWS was used regularly by residents, with 96% reporting having used it at least once while it was installed, and 45% of residents making use of it every single day (regularity of use). Most residents (59%) reported on days that they used the AHWS, they used it multiple times (frequency of use).

The AHWS represented a new form of technology. The attractiveness, innovation and novelty factor were the most significant reasons why residents used the AHWS, followed by hygiene-related reasons (see Table 4). The location of the AHWS at a transport hub in an accessible area with a considerable foot traffic also played an important role.

**Table 4:** Main reasons for choosing to use the Autarky handwashing station. Multiple answers possible.

Category	% of respondents (n_b_ = 71)	Responses
Attractiveness and novelty factor	47%	“It intrigued me” “I wanted to test and experience it” “I liked it and wanted to try it” “It had a mirror” “I loved the look of it and its structure”
Hygiene-related reasons	27%	“It had soap” “The tap water attracted me” “I needed to wash my hands”
Location	16%	“It is close to where I live” “On my way home from work” “Close to washing place” “In a good location with many passersby”

Other reasons listed by residents were that it was easy to use after going to the toilet (12%), and because the AHWS was clean and the water was clean (9%).

Residents predominantly used the AHWS after using the toilet (90%), before eating food (20%), after handling waste (12%) and when they returned home from being outside (11%) (see Fig. 4c). A range of activities were raised which were particular to the use of the AHWS, including washing hands on returning home, after sport training, after personal grooming, after buying things on the street and when passing by (see Fig. 4c). This means that the presence of a dedicated AHWS increased the reasons for washing hands and made it more convenient. Many residents particularly appreciated the mirror and indicated that the system was not only used for handwashing, but also for personal grooming.

### 4.3 Novel design and good functionality were appreciated by the users

Research on the social acceptability of the system revealed that both design and functionality shaped resident's response to the system (Table 5). Community members were drawn to use the AHWS as a result of its innovation, provision of soap, and additional design features, such as the mirror, which made the system appealing. The majority of respondents (86%) stated that they did not have any problems using the AHWS, with most stating that it was easy to use once the tap process became familiar. Residents stated that they reported problems with the system to the community leadership, the CLO or the field engineer and that the relatively few issues that arose were addressed timeously.

**Table 5:** What residents liked about the Autarky handwashing station (multiple answers possible).

Category	% of respondents (n_b_ = 71)	Responses
Soap	65%	“Having access to soap”
Mirror	30%	“It had a mirror”
Water quality	23%	“The water was clean”
Location	21%	“On my way home”
Recycling of water	11%	“The system recycles water”
Design	10%	“Loved the design”
Technology	9%	“Interesting and amazing new technology and treatment system”
Self-closing tap	9%	“You do not need to open the tap”
Water availability	9%	“Backup when basins are not working in the CAB”
Hygiene	7%	“It cleans germs and uses disinfectants”
Encouragement for handwashing	7%	“It encourages you to wash your hands”

Residents who used the system, were asked to comment on the particular elements of the system, including their design and how well they worked. This included the soap, soap dispenser, taps and sink. The respondents stated that the tap, sink and soap worked well and were easy to use for adults, but that the height of these three elements were challenging for children. A child-sized handwashing station could be developed in the future, particularly if the system is to be used in primary schools or step-up blocks could be attached to the base of the unit, in line with one of the taps for children. The details of these results are presented in the Supplementary information section (SI C).

### 4.4 Collaborative engagement was critical to the testing process

**Fig. 5:**
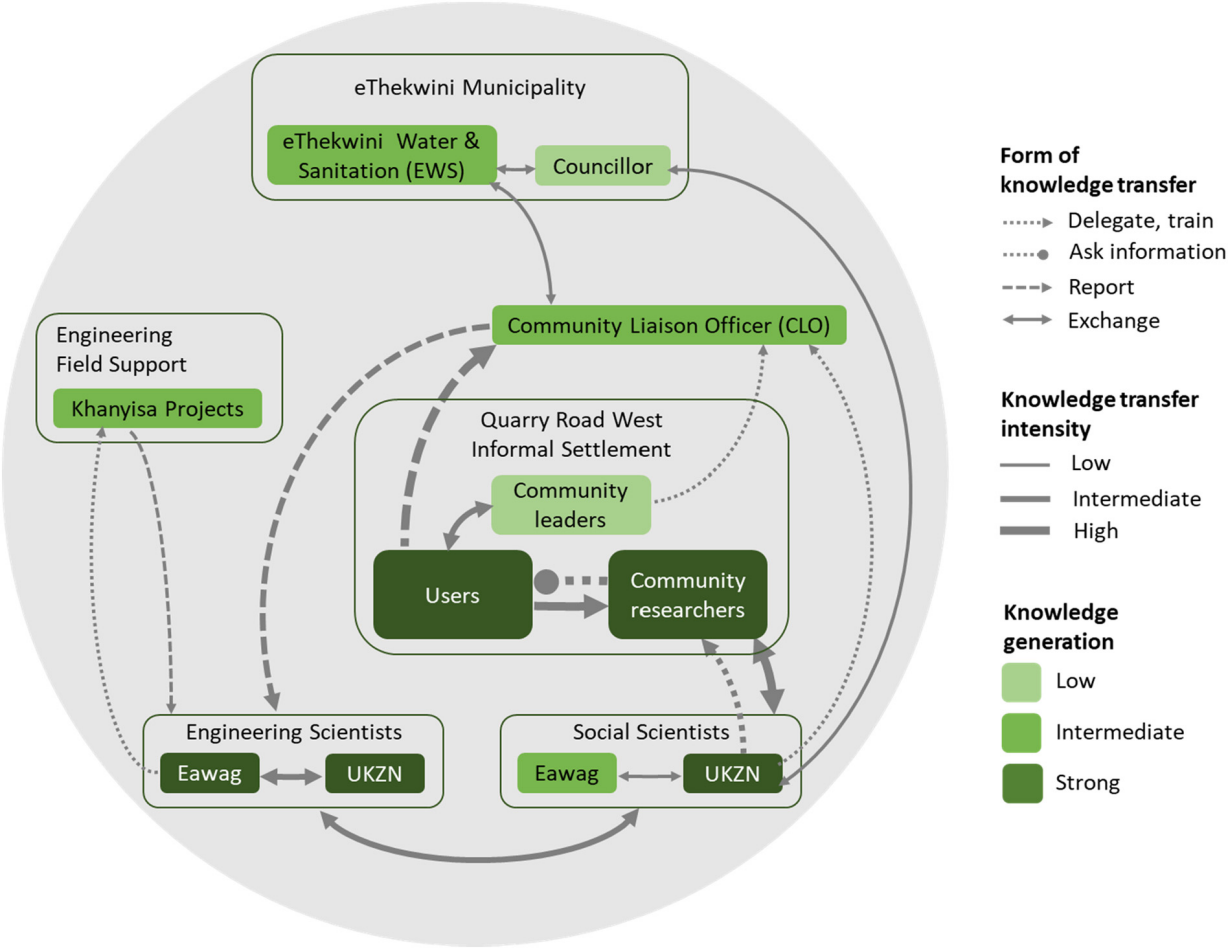
The co-production of knowledge in the testing of the Autarky handwashing station.

The Td research approach ensured that community members in Quarry Road West informal settlement formed a central part of the research process. Community members were included in a focus group to describe and discuss concerns about the testing of the AHWS before it was installed in their community. Approval to test the system was obtained from the community leadership in consultation with all residents of the settlement. Community members were included in all research processes and worked with UKZN researchers in collecting data on water and sanitation practices pre and post the installation of the system. The CLO, who was selected by the community, was appointed through Khanyisa Projects to monitor the system from a socio-technical perspective in the community and alert the engineers when issues arose. The CLO provided information to community members on how the system worked and on how to use the taps. The different stakeholders who were included in the co-production of knowledge and their level of contribution are shown in Fig. 5. The users, community researchers, UKZN social scientists and UKZN and Eawag engineering scientists contributed the most knowledge to the implementation and evaluation of the system. The strongest relationships in the co-production of knowledge around the testing of the system were between the social scientists, engineering scientists and the community. The CLO and community researchers played an important role in transferring and obtaining knowledge from users in the community.

The benefits of including community members in the testing process from the outset and their investment in the system was evident in at least two important ways during the testing process. Firstly, discussions were held between the engineers and social scientists prior to the installation of the system as to whether the AHWS should be secured using a security gate at night to stop people stealing or vandalizing the steel sink, the mirror and the soap dispenser, as these security risks are experienced in CABs. The social scientists argued that if the community were included in the process of establishing the AHWS in Quarry Road West informal settlement and in determining its location, this would not be necessary, as the community would feel a sense of ownership of the system. A decision was made in consultation with the community leadership that no security measures would be used to secure the system. Over the three months that the AHWS was present in the community, no elements of the system were stolen or damaged. Secondly, five days after opening the AHWS for use (22 April 2019), a major flood devastated Quarry Road West informal settlement. Protests were held a week later to pressurize the municipality to respond to the poor living conditions, lack of adequate services and the flood disaster in the settlement. Municipal services were vandalized during the protests. However, no damage was done to the AHWS. This was highly symbolic as it revealed the commitment of the community to the testing of this system. They felt included in the establishment and testing of the system, wanted to be part of leaving a legacy in improving WASH technologies, and hence they did not target the AHWS in the protests.

Trust is critical in ensuring the social acceptability of new technologies and part of building trust is good communication and transparency about how systems work. The residents of Quarry Road West informal settlement trusted information on the AHWS from the field engineers (27%), the UKZN researchers (27%) and those who had used recycling systems before (16%). They had limited trust in knowledge transferred to them from the municipality, which reflects the politics of and tensions between the state and citizens in service provision in the city. Media and learning about recycling at school played a role (8%), but was not dominant. This data reveals the importance of trusted knowledge brokers, such as universities, in scaling up innovative water and sanitation solutions.

**Fig. 6:**
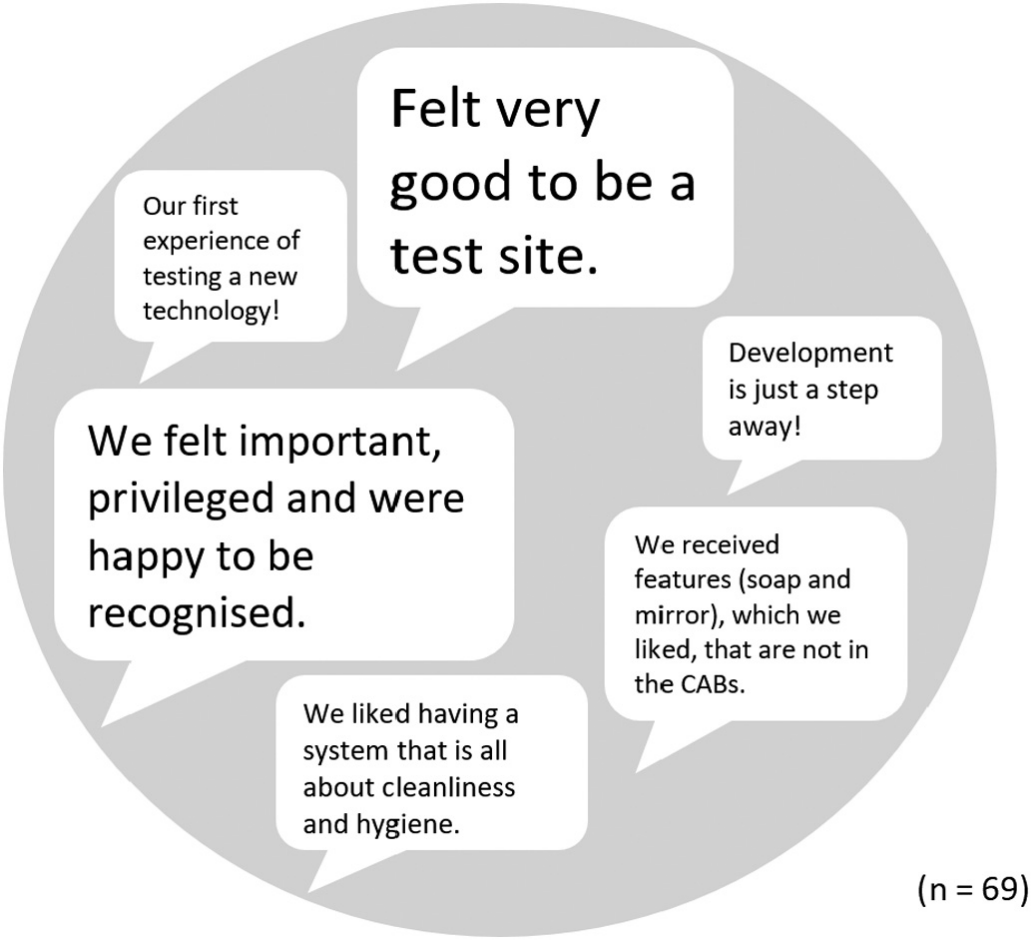
Reasons for the high level of satisfaction of being part in the testing process of the Autarky handwashing station. The text size reflects the frequency of responses.

Residents were asked how they felt about being test site for the AHWS. They were very happy to test the technology in their settlement (see Fig. 6). Respondents stated that being part of the testing enabled them to participate in the development of new technology in the city to address water and sanitation challenges, which are of major concern to them. They appreciated being recognised and included, which reflects their positionality in a city that has both progressive and repressive views about informality (Sutherland et al., 2016).

The majority of respondents (56%) stated that they were given enough information on how the water treatment process worked, while 39% said they were not given enough information and 6% reported they did not know. The majority of the respondents (59%) wanted to have more scientific knowledge on the system (Table 6).

**Table 6:** What information the respondents would like to have on the Autarky handwashing station (AHWS, multiple answers possible).

Category	% respondents (n_b1_ = 37)	Responses
How the water treatment works	49%	“To understand how they clean the water” “I need to know what happens to the dirt and germs in the water” “The whole recycling process: where the water comes from and how the machine cleans it”
Safety of the water	18%	“Is it safe to use the recycled water” “Healthy-wise information”
Field testing programme	11%	“Why they installed it here and how it works”
Importance of water recycling	8%	“Why it is important to recycle water” “What is unique about recycling water”
Rules for using the AHWS	5%	“I need to know why I am not supposed to throw water out of the machine”
Nothing	5%	“I do not need more information”

Residents are interested in the science and technology of water and sanitation services. They would like to know how they work (from beginning to end), why they are being tested in the municipality and how they should use them. This reveals that they are not passive recipients of sanitation services, but rather engaged citizens who would like to be included in the knowledge processes of water and sanitation services and that they take responsibility for their own safety, health and well-being.

### 4.5 Equality in service provision was key for the positive testing outcomes

The outcomes of the testing process revealed that the AHWS addressed several elements of equality in service provision. The technology has been tested and used in both a public setting in Zurich, Switzerland, and in an informal settlement in Durban, South Africa, which reveals its appropriateness across a range of socio-economic and development contexts. The quality of the system and its high level of innovation and design were appreciated by the informal settlement residents (see Table 4, Table 5), whose usual experience is that of being provided with services of a lower quality. Attention to detail in the design, for example the provision of a mirror and a steel sink (see Table 4, Table 5), addressed the issue that is so prevalent in South Africa, where environmentally innovative technologies are perceived to be inferior technologies that are distributed to the poor and marginalised, who are still making their way up the ladder of accessing sanitation services (Swilling, 2006). The most important aspect of addressing equality of service provision was the participation of the urban poor in the technology development process. This ensured that they had a voice in the testing of systems that could be rolled out in their settlements in the future if they are successful, and it meant that they formed part of the knowledge production and development process.

## 5 Discussion

The broader dimensions of sustainability are used to assess the value of the AHWS field test in a context of transforming and scaling up access to WASH services that are socially acceptable, environmentally sustainable and financially viable, and which are based on good governance.

### 5.1 Social sustainability

The AHWS had a high level of social acceptance in Quarry Road West informal settlement, with residents stating that they wished it could become a permanent feature. A community member made a plea for its return, during the first hard lockdown of the COVID-19 pandemic, stating that “I miss the Eawag machine in Quarry Road, *xa kunje*^4^” (Community member, 03/04/2020).

Ambole et al. (2016) and Roy (2011) argue for the participation of community members in design interventions that support informality and recognise the opportunities it provides. Local or tacit knowledge ensures that interventions are appropriate, socially just and sustainable. In this way, technological interventions become part of longer-term transformation, as a result of the knowledge capacity that is built across all stakeholders. This was the case in the testing of the AHWS, as all actors in the process learnt about innovative technology, its place in and relevance to informal settlements and the importance of collaboration.

Understanding the “cultural and historical context, needs, perception and preferences of households” (Santos et al., 2011) was important in the decision to test the AHWS in the settlement in the first instance, and in determining where it would be located to ensure its use during the period of testing and in addressing issues of ethics and social justice. This is why a participatory co-engagement process was used in testing the system. The AHWS was well located because of the community engagement in determining the site. It was highly visible, as it stood on a main transport route, at a point where individual mobility changed from buses and taxis to walking into the settlement. The AHWS acted as a niche intervention, as it met specific needs of the community and was conveniently located, while also meeting the context specific desires of the community in terms of the provision of reliable and high quality services, which the community played a role in establishing. The system was a success, as the designers focused attention on informality, thereby contributing to urban solutions in the context of rapid urbanisation in the Global South (Ambole et al., 2016).

The urban poor often perceive the implementation of innovative technology in low-cost housing projects, townships and informal settlements in South Africa as being a cost saving strategy of providing inferior technology to the poor, that middle- and upper-income groups would not accept. This results in resistance to solar water geysers, urine-diversion dehydration toilets and non-sewered sanitation systems (Bond, 2019; Swilling, 2006). What was surprising and positive in terms of the testing of the AHWS in Quarry Road West informal settlement was that it was not perceived as inferior technology, but rather as high-level innovative technology. The community was interested in using the system once it was launched, giving it the nickname of ‘the ATM’,^5^ as a result of its design features. It also inspired hope, as the project was undertaken by the municipality. Residents felt that development was finally coming to their settlement.

The residents' positive response are due to the high quality of materials used, the design features, the explanation and hence understanding of how the technology worked, the testing process, which ensured that community members could engage with and give their response to the system, and the collaboration of multiple stakeholders in an open and transparent process to assess its acceptability.

### 5.2 Environmental sustainability

The AHWS not only considered principles of social sustainability and governance, but also addressed elements of environmental sustainability. Community members are very aware of water scarcity in Durban. As the AHWS saves water and provides handwashing water when the municipal system fails, it was rated as a valuable technology for informal settlements. At the same time, they raised concerns about the safety of the water in terms of pathogens and viruses, wanting to know how they could be sure that pathogens are inactivated. This supported a co-engagement process in the testing of the AHWS, as residents wanted to engage in the science of the technology to make sure they understood how the system worked, which is important in the future scaling up of water and sanitation technologies.

Contrary to the common assumption that the urban poor are less concerned about the environment, environmental benefits were raised by residents of Quarry Road West. The main reason why residents thought water recycling is important was water scarcity, with almost all respondents indicating that saving water by using treated recycled water for handwashing water is an important need in the settlement.

Interestingly, none of the respondents mentioned the second major environmental advantage of the handwashing station: the onsite treatment of wastewater. Research on the relationship between the community and the Palmiet River has been conducted through the PCRP and residents are aware of the impact of poor services in the settlement on the river. Through the CABs, Quarry Road West informal settlement is connected to bulk infrastructure, but the removal of wastewater in the settlement is a major problem, as there are no formal drainage systems, other than those that service the CABs and communal taps, and these regularly overflow in to the settlement. Unmanaged wastewater in the settlement pollutes the environment and impacts on people's health and mobility.

### 5.3 Economic and operational sustainability

The field test of the AHWS showed that this technology could provide safe and appealing recycled water in a real-life implementation. In the context of Quarry Road West informal settlement, there is the mandate of eThekwini Municipality to provide informal settlements with water services on the one hand and the willingness of the residents to use novel water recycling technologies on the other. While it may not be realistic to implement high-technology systems in private applications (e.g., households), such WASH services could be made available in public locations such as schools, clinics or informal settlements, or all other contexts in which operation and maintenance can be ensured.

The flexibility of the system, namely easily installed, mobile, independent from large infrastructure networks and serving a number of people at the same time, makes it highly appropriate for informal settlement contexts. A local service provider can install this system quickly (and remove it again if the local situation changes). The system can be implemented where residents need a handwashing station i.e., also inside a settlement, and not only at its edges where the main infrastructure is.

The AHWS tested in Quarry Road West informal settlement is a prototype, and is currently not fit for large-scale implementation due to high construction costs and need for optimization of system components for long-term use. Key factors will be to increase the system robustness and decrease the power requirements. Before larger-scale implementation, the technology will thus need to undergo an engineering redesign to allow for mass production using modern industrial methods. When mass-produced, the cost for manufacturing such small-scale water reuse technologies can presumably be kept at a relatively low level, especially if compared to capital-intensive centralized treatment approaches (Massoud et al., 2009; Wilderer and Schreff, 2000).

Implementing water reuse technologies alone will not ensure long-term access to safe water. Operation and Maintenance (O&M), and the governance of O&M, are crucial elements of sustainability. The absence thereof is a frequent cause of failure of WASH service facilities (Barreto-Dillon, 2020), and the affected population may lose access to facilities that serve their basic needs. In the development of the AHWS, reducing the need for O&M was one of the core requirements, which was possible through the implementation of a range of technological innovations including the use of an ultrafiltration membrane supported by gravity, granular activated carbon, and the use of electrolysis to produce chlorine from the water (see SI A).

However, the testing of the AHWS also showed that technology can and does sometimes fail. During the field tests, these failures were noticed quickly, as a field engineer was on site to take water samples and to do regular system checks. Designing technologies for low maintenance is important but is not sufficient for systems where users could potentially get sick from using insufficiently treated water. If such decentralized water recycling technologies are to be implemented at a larger scale, it will be necessary to implement monitoring systems that provide real-time information on the system functioning and allow for direct intervention in case of treatment failures (Reynaert et al., 2021; Sharvelle et al., 2017). The need for such a monitoring system was acknowledged and requested by EWS, as well as community members.

The field test in Quarry Road West informal settlement revealed that the residents wanted to know how the technology works and were aware of potential risks linked to the use of recycled water. This interest could be used to improve operation and build user's trust in novel technologies. In addition to an automated monitoring system, which measures parameters that may be difficult for non-scientists to interpret, there could be benefits in providing local residents (“citizen scientists”) with simple means to check the water quality. In the case of the AHWS, this could for instance be colorimetric tests for measuring the free chlorine concentration that could provide direct and easily interpretable information on the hygienic water quality. While citizen science is increasingly used in water quality monitoring, for instance to estimate the quality of freshwaters (Capdevila et al., 2020), we would only recommend such a measure as a complement to an automated monitoring system.

In the testing of the AHWS, the combination of a local and trusted CLO on site and a technician passing by twice per week proved successful, as problems were reported quickly and the trained technician had sufficient knowledge on how these could be solved. However, this reflects exceptionalism, as maintenance of water and sanitation services becomes challenging in cities that are rapidly urbanising. EWS uses community-based caretakers to maintain the CABs and so this approach is already established in the city. However, the quick turn-around time for maintenance may become a challenge.

## 6 Conclusion

The testing of the AHWS integrated technology with real-world contexts and challenges. The Td approach adopted enabled the participation of multiple stakeholders with different knowledge systems in the testing and evaluation of the system. The research revealed that the AHWS was a niche intervention, as it met a range of community needs, including hand hygiene and personal grooming, in an equitable way, filling a gap in water and sanitation provision. The system was well-located and easy to use, convenient, and supported people's desire to practice good hand hygiene. The AHWS met higher-level social needs of equality and belonging, as residents appreciated having a robust, innovative and attractive system in their neighbourhood. Community members benefited from the co-engaged social learning process, as they were included as researchers in the assessment of water and sanitation practices in the settlement and the assessment of the system, which in turn led to greater community investment in the sustainability of the system and improved knowledge of water and sanitation challenges in their settlement. The AHWS is a valuable niche intervention for informal settlements, and would be appropriate in rural areas and schools in the Global South. The next challenge is to scale up industrial production of this technology and make it commercially viable for implementation both in the Global South and North.

## CRediT authorship contribution statement

**Catherine Sutherland:** Conceptualization, Methodology, Investigation, Formal analysis, Writing – original draft, Writing – review & editing, Visualization, Supervision. **Eva Reynaert:** Conceptualization, Methodology, Investigation, Formal analysis, Writing – original draft, Writing – review & editing, Visualization. **Rebecca C. Sindall:** Conceptualization, Methodology, Writing – review & editing, Supervision, Project administration. **Michel E. Riechmann:** Investigation, Writing – review & editing, Visualization. **Fanelesibonge Magwaza:** Investigation, Writing – review & editing. **Juri Lienert:** Investigation, Writing – review & editing. **Sibongile Buthelezi:** Investigation. **Duduzile Khumalo:** Investigation. **Sifiso Dhlamini:** Writing – review & editing. **Eberhard Morgenroth:** Writing – review & editing, Supervision. **Kai M. Udert:** Writing – review & editing, Supervision, Project administration, Funding acquisition.

## Declaration of competing interest

The authors declare that they have no known competing financial interests or personal relationships that could have appeared to influence the work reported in this paper.

## Appendix A Supplementary data

Supplementary material Image 1

## Footnotes

3 A spaza shop is an informal local or household shop, selling everyday goods to neighbours or passers-by.

4 'Xa kunje' means: when it is like this.

5 ‘ATM’ is the name used to describe automatic money machines in South Africa.
