# Supplementary material for: Innovation for improved hand hygiene: Field testing the Autarky handwashing station in collaboration with informal settlement residents in Durban, South Africa
Source: Sci Total Environ. 2021 Nov 20;796:149024. doi: 10.1016/j.scitotenv.2021.149024 (PMC8607321; doi:10.1016/j.scitotenv.2021.149024)
Supplement: Supplementary file 1 — Supplementary material [file mmc1.pdf]

Supplementary Information for

Innovation for improved hand hygiene: field testing the Autarky handwashing station in collaboration with informal settlement residents in Durban, South Africa

Catherine Sutherland[a,1], Eva Reynaert[b,c,1,*], Rebecca C. Sindall[d], Michel E. Riechmann[b], Fanelesibonge Magwaza[a], Juri Lienert[b,e], Sibongile Buthelezi[a], Duduzile Khumalo[a], Sifiso Dhlamini[d], Eberhard Morgenroth[b,c], Kai M. Udert[b,c]

https://doi.org/10.1016/j.scitotenv.2021.149024

[a] University of KwaZulu Natal, School of Built Environment and Development Studies, 4041 Durban, South Africa

[b] Eawag, Swiss Federal Institute of Aquatic Science and Technology, 8600 Dübendorf, Switzerland

[c] ETH Zürich, Institute of Environmental Engineering, 8093 Zürich, Switzerland

[d] University of KwaZulu Natal, WASH R&D Centre, 4041 Durban, South Africa

[e] Fraunhofer Ernst-Mach-Institute (EMI), 79104 Freiburg, Germany (current address)

[1] CS and ER contributed equally to this work.

*Corresponding Author: eva.reynaert@eawag.ch

The supplementary information (SI) contains the following:

SI A. provides additional information on the water recycling technology (Water Wall) used in the Autarky handwashing station.

SI B. provides details on the methods used for the water quality monitoring.

SI C. provides additional information on the informal settlement's responses to the design and functionality of the soap dispenser, tap and sink in the Autarky handwashing station.

Supplementary Information A (SI A)

The Water Wall technology uses a multi-barrier approach, with four treatment stages ensuring that the water is safe for reuse. The first barrier is an aerated bioreactor, in which active biomass is responsible for carbon oxidation, nitrification and denitrification. As a second treatment barrier, the water is filtered by gravity through an ultrafiltration membrane. The combination of bioreactor and membrane is called a biologically activated membrane bioreactor (BAMBi). The filtered water is stored in a clean water tank (CWT). In the CWT, a granular activated carbon filter acts as a third barrier, removing remaining organic contamination by adsorption and biological degradation. Finally, the fourth treatment barrier, an electrolysis unit, further reduces organic carbon concentrations and produces a chlorine residual, both of which help to limit pathogen growth during storage. This setup ensures that the Water Wall technology is capable of reliably producing safe water at low operational and maintenance costs, providing recycled handwashing water for public settings.

Supplementary Information B (SI B)

**Parameter**	**Method and/or instrument**
Chemical oxygen demand (COD)	Merck Spectroquant COD kits (0-1500 mg/L, Merck, Darmstadt, Germany)
pH	Benchtop meter (Sension MM374, Hach, Loveland, USA)
Residual chlorine	Portable spectrophotometer (DR 1900, Hach, Loveland, USA) with corresponding test kits (DPD, 0–2 mg/L free chlorine, Hach, Loveland, USA)
E. coli	Enzyme activity test (Colilert-18/Quanti-Tray, IDEXX Laboratories, Westbrook, USA)
Turbidity	Turbidimeter (2100Q, Hach, Loveland, USA)
Total suspended solids (TSS)	APHA standard method (APHA, 2005)

Supplementary Information C (SI C)

Residents of Quarry Road West informal settlement's responses to the design and functionality of the soap dispenser, tap and sink of the Autarky handwashing station.

1. Soap and soap dispenser

Table 1 presents the responses concerning the soap and soap dispenser. The majority of respondents (91%) stated that the soap dispenser was easy to use, with a resident commenting that she "wished we could have this soap in the CABs, as having soap available really helps the community" (Respondent 23, 08/04/2019). Residents that found the soap dispenser challenging stated that the soap did not come out, that you had to press the dispenser to obtain soap, which would be of concern in relation to the transmission of COVID-19, and that sometimes only a small drop came out.

Table 1. Response to the soap and soap dispenser (multiple answers are possible, n_b=71)

Response to the soap	% of sample	Response to the soap dispenser	% of sample
It was fine	56	It works well, it always worked	88
It is normal soap	13	It was easy to use	5
It made my hands fresh	11	It gave enough soap to rinse our hands well	3
It kills germs and it is safe	7	It is like the ones they have in the malls	3
It is good liquid soap	6	Nothing	3
It had a nice scent/smell	3	The button did not always work well, you had to push it a few times to get enough soap	2
It was enough soap to wash our hands	3	You do not know if there is still soap inside	2
It was weak soap but it did make a difference	3		

Table 2 presents suggested design changes to the soap dispenser. The majority of residents (88%) did not suggest any changes to the soap and soap dispenser. Of those that responded to this question, the main issue was around the design related to children's use of the system.

Table 2. Design changes to the soap and soap dispenser (multiple responses possible, n_{b2}= 24)

Design changes	% of respondents
It had all the features, it worked well, no changes needed	30
Make it lower so children can reach it	17
It is good that it is higher so children cannot break it	13
To have the dispenser show if the soap is finished inside	8
Do not know how to change it	8
Ran out of soap easily, it needs to be bigger	4
Prefer one long button to press for soap to come out rather than lots of small pushes	4
To be at the end of the handwashing station basin not above it	4
I liked it as it was something I had not used before	4
Make the soap come out automatically	4
Change the height between the tap and the soap	4

2. Tap system

The majority of residents responded positively to the tap (Table 3). They reported that the tap was easy to use (91%) and that there was always water available from the tap (94%), with 4% reporting no water, which may have been as a result of the way they used the taps, as the system always had water or they used it during the time when the system was not active.

Table 3. Response to the tap (multiple responses possible, n_b = 71)

Response to the tap	% of sample
It worked properly, it was good and easy to use	66
Unique, different and very smart	9
It was hard to use, I was not familiar with it	8
Good for saving water, controlled water use	8
It was not good as it limited how much water you could use	5
You had to push it many times, this was inconvenient	3
Tap was too short	2
Remove the electricity in the system	2

Just under one quarter of respondents commented on challenges associated with using the tap (Table 4).

Table 4. Challenges associated with using the tap (n_{b3} = 17)

Challenges with using the tap	% of respondents
No challenge there was always enough water	24
The tap water came out easily, just push it and the water comes out	24
Hard to wash your hands when you keep having to push the tap	18
No familiar with the tap, someone explained it then easy to use	12
It was too far away to use	6
At first it was difficult and then it became easy to use	6
I went to the workshop before it was installed so knew how to use it	6
Other	4

The majority of users (96%) stated that the tap provided enough water to wash your hands, with almost a third of respondents commenting on the quantity of water available (Table 5).

Table 5. Handwashing water requirements (n_{b4} = 21)

Explanation of there being enough water to wash hands	% of respondents
There was enough water, you do not need a lot of water to wash your hands	52
When you keep pressing the tap the water keeps coming out	19
The water never ran out	10
There was not enough water to wash your hands	10
Other	9

The majority of residents (81%) stated that they would not make changes to the design of the tap, while 19% suggested some changes (see Table 6).

Table 6. Design changes suggested for the tap (n_{b5} = 27)

Suggested design changes for the tap	% of respondents
Perfect as it is, had all the features including a mirror, provided enough water	37
It was designed like this to save water	15
Rather make the water come out automatically with a sensor	11
Use a button tap	7
If it had been a normal open and closing tap people would have broken it	7
It needs to be lower for children	7
Make the tap more comfortable	4
Make it plastic to avoid it giving shocks	4
It should be a normal tap that opens and closes in a normal way	4
Other	4

3. Sink/Basin

The majority of users were happy with the design and function of the sink or basin (Table 7).

Table 7. Response to the sink (multiple responses possible, n_b = 71)

Response to the sink	% of sample
It was fine	62
It is a normal sink	6
It is good for adults and children to wash their hands in	4
Would be better if it was plastic as that would means it could not shock you	4
Need more space between the system and other people, or people standing using the sink	3
It worked well in terms of water flowing in to it, water did not spill out of it so water was not wasted	3
It had dust on it	3
It was wide enough to wash your hands	3
It was very small	3
It was clean all the time	3
It was long	2
It should be more shallow so that no water is wasted	2
It could be harmful to children	2

Just under a third of respondents made suggestions about the design of the tap, with 17% stating that they would make changes to it, as reflected in Table 8. Respondents (43%) liked the tap and indicated that it had all the features they needed.

Table 8. Suggested design changes to the sink (n_{b6} = 21)

Suggested sink design changes	% of respondents
No change, it is perfect	42
It should have a longer basin	14
Have a plastic sink so that it cannot shock you	14
They need to put a net or wire where the water goes down the sink	5
Lower it a bit or have steps for children	5
Make it bigger between the person and the tap	5
No enough space between tap and the sink	5
Change it to stainless steel so it does not rust	5
It should be shallower	5

4. Height of soap dispenser, tap and sink

Figure 1 summarizes the responses to the height of the soap dispenser, tap and sink for adults and children (n_b = 71).

Height of Soap dispenser

Acceptable: Adults: 94% Children: 39%

Not acceptable: Adults: 4% Children: 59%

No response: 2%

Height of Tap

Acceptable: Adults: 96% Children: 35%

Not acceptable: Adults: 4% Children: 58%

Don’t know children: 7%

Height of Sink

Acceptable: Adults: 96% Children: 41%

Not acceptable: Adults: 4% Children: 53%

No response children: 6%

Figure 1. Response to height of different elements of the system: soap dispenser, tap and sink
